# Chronic patients’ satisfaction and priorities regarding medical care, information and services and quality of life: a French online patient community survey

**DOI:** 10.1186/s12913-020-05373-5

**Published:** 2020-06-05

**Authors:** Apolline Adé, Frédérique Debroucker, Laura Delporte, Cécile De Monclin, Emmanuel Fayet, Pierre Legendre, Lise Radoszycki, Michael Chekroun

**Affiliations:** 1 Medtronic, Boulogne-Billancourt, France; 2 Carenity, 1 Rue de Stockholm, 75008 Paris, France

**Keywords:** Chronic conditions, Patient community, Real-word insights, Satisfaction, Priorities, Medical care, Information, Services, Quality of life

## Abstract

**Background:**

The French healthcare system is evolving to meet the challenges of an aging population, the growing prevalence of chronic diseases, the development of new technologies and the increasing involvement of patients in the management of their disease. The aim of this study is to assess the satisfaction and priorities of chronic patients regarding medical care, information and services and their quality of life.

**Methods:**

A cross-sectional study was conducted from February to March 2018 via the French Carenity platform. Adult patients enrolled in type 1 diabetes, heart failure or obesity communities were invited to answer an online questionnaire. A numeric scale from 0 (meaning not satisfied) to 5 was used to evaluate patients’ satisfaction. Patients’ priorities were assessed using a ranking question. Patients’ satisfaction and priorities have been combined in a matrix to identify patients’ expectations.

**Results:**

Sixty-seven respondents of each condition answered a questionnaire. The most important and least satisfactory items about medical care are availability and active listening from healthcare providers, as well as access to coordinated and multidisciplinary care. Regarding information and services, respondents mostly expect connected medical devices, in addition to lifestyle and dietary measures. As for the quality of life, respondents fear that their chronic condition will keep impacting their daily mood and ability to do physical activities.

**Conclusions:**

This study shows that chronic patients want to be more actively involved in their care pathway. Patient training and therapeutic patient education programs could help them manage their chronic conditions within a patient-centred healthcare system.

## Background

Healthcare systems in developed countries were created to manage acute diseases rather than chronic conditions and are no longer suited to an environment that has radically changed due to a triple transition: demographic, epidemiologic and technologic. The demographic and the epidemiologic transitions are explained by an aging population and an increase in the prevalence of chronic diseases [1] and will produce an increase in healthcare expenditures. The technological transition is marked by the development of new information and communication technologies such as telemedicine or connected devices and supports the shift from acute care to chronic care. The emergence of digital technologies makes an efficient coordination of healthcare providers possible and contributes to the development of patient-centered care by encouraging patients to participate in their own health. The success of a new healthcare system depends on the promotion of patient involvement in their own care management and the development of care pathways.

The concept of integrated care, or care pathways, appears to be a solution to meet the care and support needs of patients affected by chronic diseases [2, 3]. The care pathway is based on patient-centered care and requires coordination and cooperation among healthcare professionals [4]. Active patient participation is a key factor for the successful management of chronic conditions. It requires identifying and integrating patient needs and preferences into decisions regarding health practices [5]. This study aimed to assess the needs and preferences of patients with chronic conditions. This is an essential preliminary step that will contribute to addressing patient needs and preferences through actions.

Patients and their caregivers share more and more information on various online platforms about health topics and their own experience. It offers new research possibilities to identify and better understand patient needs and patient-reported outcomes about care and support [6–8]. Online platforms also provides access to patients’ satisfaction and preferences in several diseases [9, 10], thus this study targets other chronic patients who share their stories on an online patient community. A virtual community in healthcare has been defined as “a social unit that involves members who relate to one another as a group and interact using communication technologies that bridge geographic distance” [11]. The study focused on type 1 diabetes, obesity and heart failure because their prevalence is high in France and because they embody major public health challenges. The primary aim is to assess the French online community chronic patients’ satisfaction and priorities concerning medical care, information and services and the impact of the disease on the quality of life.

Medtronic is embracing the shift to value-based healthcare. Medtronic France ordered the launch of this study because, as a Medtech company, Medtronic supports efforts to drastically restructure healthcare delivery systems and make payment for products and services contingent upon the ability to improve patient outcomes relative to the cost [12].

## Methods

### Study design

Carenity is a free online international patient community with more than 400,000 patients and caregivers and 1200 chronic conditions across Europe and the United States [13]. Carenity provides patients with online support to help them share their health-related experiences and monitor their symptoms and treatments.

This cross-sectional study was conducted on the French Carenity platform from February to March 2018. All adult patients registered in type 1 diabetes, heart failure and obesity communities were invited, via e-mails and private messages on Carenity, to participate in a confidential online satisfaction survey. We have chosen three high-prevalence pathologies for which the role of the patient in the management is important. They are therefore patients who are sufficiently involved in their care pathways to give their opinion and priorities. The methodology was chosen to limit the bias of the interviewer (doctor or investigator) so that patients could share their opinion as honestly as possible about their level of satisfaction with their care by answering an online questionnaire.

As members of the Carenity platform, patients participating in the study provided explicit informed consent to the collection, handling and keeping of their personal and health data. They were also provided with specific information on the goals and procedures of the study and were asked to agree to participate before starting the study questionnaire.

Data was pseudonymized and participants’ data were checked at the end of the data collection process due to absence of pharmacovigilance signal. A data quality plan was defined before fieldwork and then tested according to: (i) response speed to the survey (estimated at 15 min); (ii) coherence of answers (medical profile, coherence between questions). All patients who fully completed the survey and successfully achieved the quality check described above were consecutively included in the study. Final sample size was determined by the combination of the capability of the platform, the time allocated for data collection, sub-groups of homogeneous size, and sufficient statistical power to make comparisons (size of subgroups > 30). The study design was not modified after the launch of the project.

### Survey questionnaire

Three survey questionnaires, one for each chronic condition, were developed by Carenity. Each one was proof-read by a patient to ensure that the questions are understandable and the response options are appropriate, then approved by two Medtronic members of the Think Tank “Le cercle de la valeur en sante” (healthcare professionals, patients, payers, economists and regulators) who gave their opinion on the appropriateness of the wording of the research questions [14]. Depending on the condition, the questionnaires contained up to 31 questions with 4 open-ended questions (See Supplementary file).

If a patient were enrolled in more than one community, they were asked to answer the survey of the chronic condition that impacts their quality of life the most.

Patients’ satisfaction was assessed using a numeric scale with 0 the lowest satisfaction and 5 the highest. To evaluate what matters most to them, patients were asked to rank 5 items they consider to be the most important amongst a list of 9 items for medical care, 8 items for information and services and 8 items for quality of life. The unranked criteria were ranked 6.

Patients’ satisfaction and priorities have been combined in a matrix to identify patients’ expectations: the x-axis represents patients’ priorities and the y-axis represents patients’ satisfaction. Four levels of priority were defined on the matrix using the mean satisfaction (MS) and the priority mean rank (PMR): priority 1 (MS < median and PMR < median), priority 2 (MS < median and PMR > median), priority 3 (MS > median and PMR < median), priority 4 (MS > median and PMR > median).

### Statistical analysis

Microsoft Excel 2013 and RStudio (v3.5.0) were used to perform descriptive, univariate and multivariate analysis. When n < 30, Shapiro-Wilk test was performed to assess the normality of the distribution. The multivariate analyses of quantitative variables included Student test and ANOVA test when the population was normally distributed and Wilcoxon test and Kruskal-Wallis test when it was not. Multivariate analysis also included Chi-squared test for categorical variables.

Other results of the study are presented as means for quantitative variables and frequencies for categorical variables.

## Results

### Respondents’ profile

A total of 201 respondents answered the three surveys: 67 affected by type 1 diabetes, 67 affected by heart failure and 67 affected by obesity. Respondents’ characteristics are presented in Table 1.

**Table 1:** Respondents’ characteristics

	Global (*n* = 201)	Type 1 diabetes (*n* = 67)	Heart failure (*n* = 67)	Obesity (*n* = 67)
**Sex**				
Male	84 (41.8)	18 (26.9)	43 (64.2)	23 (34.3)
**Age**				
18–30 years	4 (2.0)	4 (6.0)	0 (0.0)	0 (0.0)
31–40 years	13 (6.5)	10 (14.9)	1 (1.5)	2 (3.0)
41–50 years	38 (18.9)	15 (22.4)	8 (11.9)	15 (22.4)
51–60 years	60 (29.9)	20 (29.9)	19 (28.4)	21 (31.3)
61–70 years	60 (29.9)	13 (19.4)	24 (35.8)	23 (34.3)
> 70 years	26 (12.9)	5 (7.5)	15 (22.4)	6 (9.0)
Age, years (mean ± SD)	57.3 ± 11.7	51.6 ± 13.1	62.6 ± 9.9	57.8 ± 9.0
**Diagnosis**				
Age when diagnosed, years (mean ± SD)	38.3 ± 18.5	25.1 ± 16.7	50.4 ± 14.2	39.4 ± 15.1
Time since diagnosis, years ago (mean ± SD)	19.1 ± 15.4	26.5 ± 16.2	12.3 ± 10.6	18.4 ± 15.5

Data are expressed as n (%) unless otherwise specified, SD standard deviation

Most respondents are treated in hospitals and clinics (59%), half of them (50%) are also treated in a medical office. Only 8% are monitored within a specialized structure and 4% in a medical center. 12% of respondents with type 1 diabetes attend a specialized structure followed by 9% of respondents with obesity and only 4% of respondents with heart failure.

New York Heart Association (NYHA) classes were used to evaluate heart failure patients’ functional ability [15]. 15% of respondents reported no limitation of physical activity (class I NYHA). Almost one in two respondents (47%) reported a slight limitation of physical activity (class II NYHA), 28% reported a marked limitation of physical activity (class III NYHA) and 10% reported severe limitation of physical activity with symptoms present even at rest (class IV NYHA).

Body Mass Index (BMI) was calculated: 36% of obese respondents were obese (30 < BMI ≤ 35), 36% were severely obese (35 < BMI ≤ 40) and 28% were morbidly obese (BMI > 40).

### Respondents’ satisfaction and priorities regarding medical care

Respondents were most satisfied with the reputation of healthcare providers (4.1/5), followed by the quality of infrastructures and services (3.2/5) and access to healthcare providers (3.2/5). On the other hand, respondents were least satisfied with relatives or other patients’ recommendation for the place of care (2.2/5), as well as access to coordinated and multidisciplinary care (2.6/5). Respondents with type 1 diabetes are more satisfied than respondents with obesity or heart failure with the access to innovative drugs and medical devices (3.8/5, 3.1/5 and 1.8/5 respectively) (p < 0.01) and with taking the patients’ opinion into account for the choice of treatment (3.9/5, 2.7/5 and 2.3/5 respectively) (p < 0.01). Respondents with type 1 diabetes are more satisfied (3.1/5) than respondents with obesity (2.1/5) with the access to coordinated and multidisciplinary care (p < 0.05). The quality of infrastructures and services in place of care is more satisfactory for respondents with type 1 diabetes (3.3/5) and heart failure (3.6/5) than for respondents with obesity (2.7/5) (p < 0.05). In general, respondents with obesity are dissatisfied with medical care. Indeed, 21% of them underwent surgery and complained about the post-surgery follow-up (1.6/5).

Availability and active listening from healthcare providers was ranked the most important criteria concerning medical care for respondents (3.2/6), followed by the access to innovative drugs and medical devices (3.6/6), access to coordinated and multidisciplinary care (3.8/6), taking the patient’s opinion into account for the choice of treatment (4.0/6) and access to healthcare providers (4.2/6). Taking the patient’s opinion into account is more important for respondents with type 1 diabetes (3.5/6) than for respondents with both obesity and heart failure (4.3/6) (p < 0.01). Respondents affected by obesity give more importance to coordinated and multidisciplinary care access (3.5/6) than respondents with type 1 diabetes (4.2/6) (p < 0.05). Recommendation of the place of care by relatives or other patients is also more important for obese respondents (5.2/6) than for type 1 diabetes respondents (5.6/6) (p < 0.05).

**Fig. 1:**
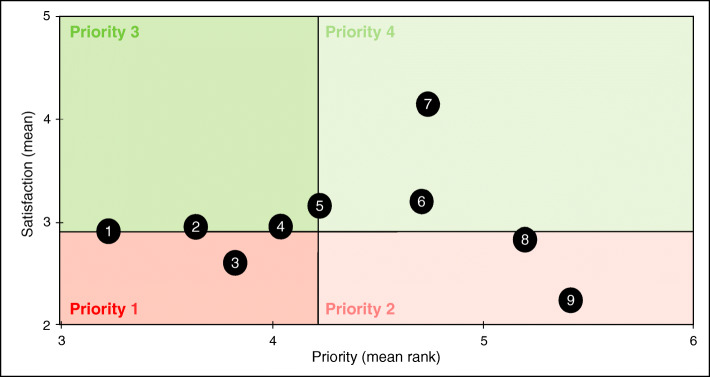
Respondents’ satisfaction and priorities regarding medical care. Figure 1 shows respondents’ expectations about medical care, crossing the mean satisfaction (range 0–5, 0 meaning not satisfied) and the priority mean rank (range 1–6, 1 meaning most important criterion). Both medians were used to create a matrix with 4 priority levels (level 1 meaning most important area). Items located in the red areas are the least satisfactory for patients. Items located in the darker areas of both the red and green areas are the most expected by patients. The 9 following items were displayed on the matrix: 1: availability and active listening from healthcare providers. 2: access to innovative drugs and medical devices. 3: access to coordinated and multidisciplinary care. 4: taking the patient’s opinion into account for the choice of treatment. 5: access to healthcare providers. 6: quality of infrastructure and services. 7: reputation of healthcare providers. 8: recommendation of the place of care by healthcare professionals. 9: recommendation of the place of care by relatives or other patients

According to the matrix, access to coordinated and multidisciplinary care, availability and active listening from healthcare providers and taking the patient’s opinion into account for the choice of treatment are both the least satisfactory and the most important items regarding medical care (Fig. 1, Supplementary Table S1).

### Respondents’ satisfaction and priorities regarding information and services

The item the respondents were most satisfied with was information and practical advice (3.2/5), followed by websites and mobile applications (2.7/5) and connected medical devices (2.6/5). The least satisfactory services are telemedicine, psychological support and connected devices (1.7/5, 2.1/5 and 2.1/5 respectively). Respondents with type 1 diabetes are more satisfied with connected medical devices (3.6/5) than respondents with heart failure (2.3/5) and obesity (1.8/5) (p < 0.01). They are also more satisfied with telemedicine (2.1/5) than respondents with obesity (1.1/5) (p < 0.05).

Information and practical advice was ranked the most important criteria for respondents (2.8/6), followed by lifestyle and dietary measures (3.7/6), connected medical devices (3.8/6), and both scientific news and psychological support (4.0/6). Lifestyle and dietary measures are more important for respondents with obesity (2.7/6) than for respondents with heart failure (4.1/6) and type 1 diabetes (4.3/6) (p < 0.01). They also consider psychological support as more essential (3.5/6) than the others (p < 0.01). Type 1 diabetes respondents give more importance to connected medical devices (2.7/6) than other respondents (p < 0.01). Telemedicine is more important for respondents with heart failure (4.3/6) than other respondents (p < 0.01).

**Fig. 2:**
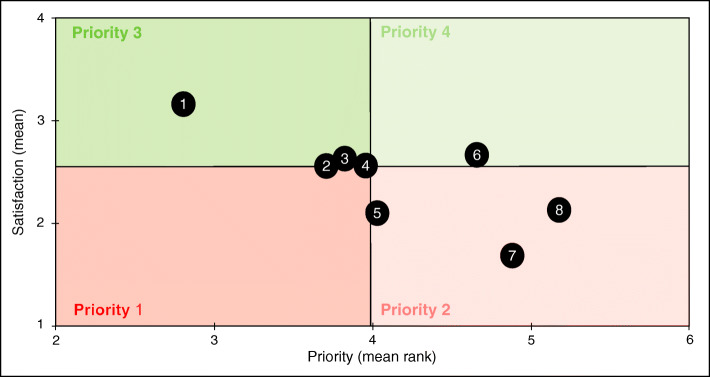
Respondents’ satisfaction and priorities regarding information and services. Figure 2 shows respondents’ expectations in terms of information and services, crossing the mean satisfaction (range 0–5, 0 meaning not satisfied) and the priority mean rank (range 1–6, 1 meaning most important criterion). Both medians were used to create a matrix with 4 priority levels (level 1 meaning most important area). Items located in the red areas are the least satisfactory for patients. Items located in the darker areas of both the red and green areas are the most expected by patients. The 8 following items were displayed on the matrix: 1: information and practical advice. 2: lifestyle and dietary measures. 3: connected medical devices. 4: scientific news. 5: psychological support. 6: websites and mobile applications. 7: telemedicine. 8: connected devices

According to the matrix, connected medical devices, lifestyle and dietary measures, and psychological support are both the least satisfactory and the most important items in terms of information and services (Fig. 2, Supplementary Table S2).

### Respondents’ perception and priorities regarding the impact of the chronic condition on quality of life

The most impacted aspect of respondents’ quality of life due to the condition is daily mood (3.5/5) followed by social and family life (3.4/5) and food choices (3.4/5). The least affected aspects are autonomy (2.2/5) and impact on friends and family (2.8/5). Respondents with heart failure and obesity consider that their autonomy is more impacted by their condition (2.6/5 and 2.5/5 respectively) than respondents with type 1 diabetes (1.7/5) (p < 0.01). Respondents with obesity and type 1 diabetes consider that their condition has a greater impact on food choices (3.7/5 and 3.6/5 respectively) than respondents with heart failure (2.9/5) (p < 0.01). Respondents with obesity consider that their condition has a greater impact on friends and family (3.1/5) than type 1 diabetes respondents (2.3/5) (p < 0.05).

Daily mood was ranked the most important aspect on quality of life for respondents (3.3/6), followed by ability to do physical activities (3.4/6), social and family life (3.6/6), autonomy (3.7/6) and food choices (4.4/6). The most important aspect of quality of life is daily mood for type 1 diabetes respondents (3.0/6) and respondents with obesity (3.1/6). It is autonomy for respondents with heart failure, followed by ability to do physical activities (3.1/6 and 3.3/6 respectively). Respondents with type 1 diabetes and obesity give greater importance to daily mood than respondents with heart failure (3.8/6) (p < 0.01). Autonomy is considered as more important by respondents with heart failure than by respondents with type 1 diabetes (4.2/6) (p < 0.01).

**Fig. 3:**
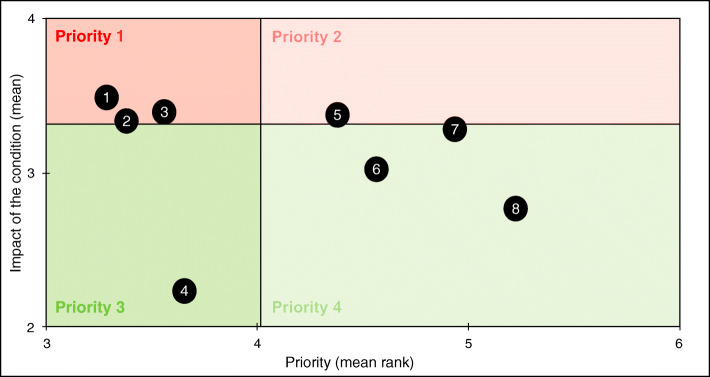
Respondents’ perception and priorities regarding the impact of the chronic condition on quality of life. Figure 3 shows respondents’ expectations regarding the impact of the chronic condition on quality of life, crossing the mean impact (range 0–5, 0 meaning no impact) and the priority mean rank (1–6, 1 meaning most important aspect to preserve). Both medians were used to create a matrix with 4 priority levels (level 1 meaning most important area). Items located in the red areas are the most impacted by the condition. Items located in the darker areas of both the red and green areas are the most important for patients. The 8 following items were displayed on the matrix: 1: daily mood. 2: ability to do physical activities. 3: social and family life. 4: autonomy. 5: food choices. 6: love/sex life. 7: professional life. 8: friends and family

According to the matrix, daily mood, the ability to do physical activity and social and family life are both the most important aspects of respondent’s quality of life and the ones that they fear their chronic condition will impact the most (Fig. 3, Supplementary Table S3).

The most important and least satisfactory aspects regarding medical care, information and services and the impact of the chronic condition on the quality of life are presented in Supplementary Table S4 for each condition.

### Respondents’ care pathways

32% of respondents reported following a therapeutic patient education (TPE) program; 42% (27/64) of them are very satisfied by their TPE (3.0/5). Respondents with type 1 diabetes are the most involved in TPE programs (42%), followed by obese respondents (28%) and respondents with heart failure (25%). Obese respondents are less satisfied with TPE programs (2.3/5) than respondents with heart failure and type 1 diabetes (3.3/5 and 3.4/5 respectively) (p < 0.05).

Respondents reported being very interested in participating in coordinated and multidisciplinary medical care, especially if it can improve the outcome of treatments (8.5/10), reduce out-of-pocket costs (7.7/10), and simplify the organization of medical care (7.5/10) (10 meaning very interested to get involved).

Most respondents (68%) use medical devices: 100% of respondents with type 1 diabetes, 55% of respondents with heart failure and 48% of respondents with obesity (p < 0.01). 59% of respondents think that medical devices vastly improve their quality of life. Respondents with type 1 diabetes consider that medical devices help to considerably improve their quality of life (78%) to a larger extent than both obese and heart failure respondents (40%) (p < 0.01).

Many respondents are willing to better carry their voice to healthcare authorities: 44% would like to become expert patients and 37% are considering joining a patient organization to be able to debate with healthcare providers and industries. 63% of respondents believe that sharing the discussions they have with healthcare professionals with health authorities would help patients’ opinion to be better taken into account. Almost one in two respondents (47%) also believe that improving access to trainings to become expert patients would allow more consideration of patients’ opinion by health authorities.

## Discussion

The results of this study underline the expectations of Carenity online community members affected by type 1 diabetes, obesity and heart failure regarding medical care, information and services and the impact of the disease on their quality of life.

Firstly, the study shows that the three most important and least satisfactory items regarding medical care are availability and active listening from healthcare providers, access to coordinated and multidisciplinary care and taking the patient’s opinion into account for the choice of treatment.

Encouraging and supporting coordinated and multidisciplinary care is relevant because multi-disciplinary care has been demonstrated to improve outcomes for patients with chronic conditions [16–18]. The study conducted by Tapp et al. showed that a multidisciplinary team approach can contribute to an improved chronic care management for diabetic patients [19]. Another solution is the active listening method, a concept developed by American psychologist Carl Rogers to help patients to clarify what works, what they can change and encourage them to set realistic goals [20]. The objective is to create a suitable environment for the patient in order to help them express their feelings as clearly as possible and develop a relationship of trust with healthcare providers [21]. In the US, advanced practice nurses are encouraged to perform active listening with obese patients because it is an excellent communication tool to promote dietary behavior changes [22].

Results of the study confirm that the former passive role of patients has developed into an active participation in healthcare. Indeed, with the ongoing democratisation of healthcare and the development of expert patient concepts and therapeutic education programs, healthcare authorities and decision makers have reinforced the active participation of patients in the design of healthcare policies. One in two patients believe that their opinion would be better considered if more patients became expert patients and participated in working groups with health authorities and industries. 44% would be willing to become an expert patient. 41% of patients believe that joining a patient organization to debate with healthcare providers and healthcare authorities would allow the patient’s opinion to be better taken into account. Since 2017, the French health authority has been inviting patient organizations to express their views on how patients experience their diseases and current treatments. The goal is to involve patients within the health technology assessment of drugs and medical devices [23, 24].

A patient-centered integrated care framework contributes to the empowerment of patients so they have a more active role in their own chronic condition. It puts the needs and preferences of patients at the center of healthcare systems. In France, article 51 (LFSS 2018) has been created to experiment with new healthcare organizations, in order to improve the patients’ journey and the efficiency of the healthcare system [25]. These innovative organizations will be supported by new funding models that could help to cover healthcare expenditures that are not currently covered or too expensive for patients. A program to address the issue of long-term follow-up of obese patients undergoing surgery is currently being developed by Medtronic France and a scientific committee. A new care pathway and a new funding model will be implemented for 5 years. The future results of this study regarding obese patients will inspire the creation of customized solutions and services.

Secondly, connected medical devices, lifestyle and dietary measures and psychological support are the least satisfactory and the most important items for patients concerning information and services.

Obese patients expect more psychological support than other respondents and patients who underwent bariatric surgery are very dissatisfied with the post-surgery follow up. This is consistent with the IGAS (“Inspection Générale des Affaires Sociales”) recommendations which include strengthening ways to improve post-surgery follow-up for patients affected by obesity and map medical and paramedical needs and resources [26].

Information about lifestyle and dietary measures can be provided for patients through TPE program. Only one third of participants reported following a TPE program and 42% of them are very satisfied with it. TPE programs have a positive impact on the patient’s health and quality of life, as well as their relatives’ [27]. Healthcare providers and the industry should enhance patient empowerment through the development of therapeutic education programs and self-management tools. The training of healthcare providers should also be reinforced to allow them to develop the necessary skills to provide TPE program. In 2015, the French Task Force on therapeutic education in heart failure published a framework to create a cross-functional structured program for therapeutic education of patients with chronic heart failure [28]. The objective of this program is to provide a reference point to facilitate the development of a personalized program for each patient. Yet, only few patients affected by heart failure have access to this kind of programs.

Participants with type 1 diabetes consider that connected medical devices are the most important service that can improve their daily life. However, most participants reported being dissatisfied with connected medical devices and patient monitoring. In order to generalize the use of connected medical devices, medical technologies companies are facing new challenges. They need to better understand the end-user and to provide data about the safety and effectiveness of the device. The design of connected medical devices should integrate patients’ preferences to address patient needs. Easy-to-use devices with intuitive interface could also help patients and physicians to better understand the added value of connected medical devices. Patients’ willingness to use technologies in healthcare is often studied with the Technology Acceptance Model [29]. In 2019, the French health authority published a guide on the evaluation of connected medical devices.

Our study shows that respondents with heart failure have higher expectations about telemedicine than patients with type 1 diabetes and obesity. This is consistent with the 2016 guidelines of the European Society of Cardiology [30]. Moreover, the Telemedical Interventional Management in Heart Failure II trial conducted between 2013 and 2017, involving more than 1500 patients with chronic heart failure, has demonstrated the benefit of structured remote patient management intervention in reducing the number of deaths and hospitalizations [31].

Finally, the most important aspects of the quality of life for respondents and the ones that they fear their chronic condition will impact the most are daily mood, the ability to do a physical activity and social and family life.

A chronic disease disrupts life and has an impact on the well-being, physical and mental health of an individual. In the DAWN2™ study, almost one in two patients experience permanent psychological distress related to their disease, feel that they have a reduced quality of life, and do not find the desired support from their family or close ones [32].

The fact that participants of our study are requesting information shows that they want to better understand their condition in order to improve its management. A similar result has been observed in the study ENTRED 2: 85% of people with type 1 diabetes were well informed about their disease but still wanted to improve their knowledge about their condition [33].

This study shows that conducting surveys on an online community is a good way to gather and identify patients’ preferences and needs. Online communities generate real-world insights that are highly valuable and complementary to clinical trial data. Furthermore, this study helped to highlight satisfaction and priorities of patients with type 1 diabetes, obesity and heart failure. These results could be the basis of a trial to develop valid Patient-Reported Outcomes Measures (PROMs).

Several limitations of this work are noted, including that all questions were mandatory that forced patients to adopt a position on questions requesting their opinion. This can induce some bias when patients do not have a clear opinion. Medical data provided by the patients was not confirmed by medical practitioners. This may potentially compromise the validity of the disease related data. Nevertheless, the objective of the study was to talk about the patient’s satisfaction and therefore this bias related to the medical profile is limited. Since this study has been conducted only with Carenity members, the results may not be fully representative of the global population. However, in 2018, Raïs et al. performed a comparison between Carenity patients with type 1 and type 2 diabetes, multiple sclerosis, Parkinson’s disease and inflammatory bowel disease and a sample of representative French patients from SNIIRAM (“Système National d’Information d’Interrégimes de l’Assurance Maladie”) [34]. They showed that Carenity communities are representative of the French population with these chronic conditions, with an over-representation of female patients, aged from 25 to 54 years old. Moreover, patients registered on Carenity are more involved in the management of their health problem, so it can be assumed that they have higher expectations of their care.

Though the sample size for each condition is small (n = 67) in comparison with the number of patients affected by these conditions in France, it allows sufficient statistical power. Moreover, patients’ satisfaction was assessed using a numeric scale from 0 to 5 because there is no specific measurement indicator to assess patients’ satisfaction regarding medical care. The creation of standardized indicators such as PROMs for patients affected by chronic diseases would be useful to measure their satisfaction throughout all stages of their care journey.

The findings of this study are powerful tools and should be broadly shared (i) to raise awareness about patient needs and preferences and (ii) to address them through actions. These findings will be useful for healthcare providers, stakeholders and patient representatives who wish to integrate patient preferences in clinical practice guidelines or in healthcare policy decisions [35–37]. The findings of the study should also stimulate creative thinking of pharmaceutical and medtech industries for developing tailored solutions and services and integrating them in experimentation of new healthcare organizations.

## Conclusions

This cross-sectional study underlines the satisfaction and priorities of Carenity members affected by type 1 diabetes, obesity and heart failure regarding medical care, information and services and the impact of the chronic condition on quality of life. The most important and least satisfactory items concerning medical care are availability and active listening from healthcare providers, access to coordinated and multidisciplinary care and taking the patient’s opinion into account for the choice of treatment. As for information and services, respondents mostly expect connected medical devices, lifestyle and dietary measures and psychological support. Concerning the quality of life, respondents fear their chronic condition will keep impacting their daily mood, their ability to do physical activities and their social and family life.

This study also shows that patients affected by chronic diseases want to be more actively involved in their healthcare management. Patient training and therapeutic patient education programs are much relevant in the management of chronic diseases and are taken into account by health authorities. The successful management of chronic conditions is based on a patient-centred healthcare system.

The transition of the healthcare system from acute care to chronic care relies on the active participation of patients in the management of their own care and in the adoption of healthy lifelong behaviors. The findings of the study are the first step towards the integration of patient preferences in clinical practice guidelines and in healthcare policy decisions.

## Supplementary information

**Additional file 1: Table S1.** Respondents’ satisfaction and priorities regarding medical care. **Table S2.** Respondents’ satisfaction and priorities regarding information and services. **Table S3.** Respondents’ perception and priorities regarding the impact of the chronic condition on quality of life. **Table S4.** The study’s main results.

**Additional file 2.**

**Additional file 3.**

## Data Availability

The datasets generated and analysed during the current study are not publicly available due to the presence of identifiable data but some parts may be available from the corresponding author on reasonable request.

## Abbreviations

BMI: Body Mass Index
IGAS: Inspection Générale des Affaires Sociales
MS: Mean Satisfaction
NS: Not Significant
NYHA: New York Heart Association
PMR: Priority Mean Rank
PROM: Patient-Reported Outcomes Measures
SD: Standard Deviation
SNIIRAM: Système National d’Information d’Interrégimes de l’Assurance Maladie
TPE: Therapeutic Patient Education
