# Supplementary material for: Chronic patients’ satisfaction and priorities regarding medical care, information and services and quality of life: a French online patient community survey
Source: BMC Health Serv Res. 2020 Jun 5;20:511. doi: 10.1186/s12913-020-05373-5 (PMC7275411; doi:10.1186/s12913-020-05373-5)
Supplement: Supplementary file 1 — Additional file 1: Table S1. Respondents’ satisfaction and priorities regarding medical care. Table S2. Respondents’ satisfaction and priorities regarding information and services. Table S3. Respondents’ perception and priorities regarding the impact of the chronic condition on quality of life. Table S4. The study’s main results. [file 12913_2020_5373_MOESM1_ESM.docx]

**Supplementary Table 1: Respondents’ satisfaction and priorities regarding medical care**

|  | | | **Global**  **(n = 201)** | | **Type 1 diabetes**  **(n = 67)** | | **Heart failure**  **(n = 67)** | **Obesity**  **(n = 67)** | **p-value** |
| --- | --- | --- | --- | --- | --- | --- | --- | --- | --- |
| Availability and active listening from healthcare providers | Satisfaction | Mean | 2.9 | 3.3 | | 2.7 | | 2.7 | NS |
|  | Priority | Mean rank | 3.2 | 3.2 | | 3.3 | | 3.2 | NS |
| Access to innovative drugs and medical devices | Satisfaction | Mean | 3.0 | 3.8 | | 3.1 | | 1.8 | *** |
|  | Priority | Mean rank | 3.6 | 3.4 | | 3.6 | | 4.0 | NS |
| Access to coordinated and multidisciplinary care | Satisfaction | Mean | 2.6 | 3.1 | | 2.5 | | 2.1 | ** |
|  | Priority | Mean rank | 3.8 | 4.2 | | 3.8 | | 3.5 | * |
| Taking the patient’s opinion into account for the choice of treatment | Satisfaction | Mean | 3.0 | 3.9 | | 2.7 | | 2.3 | *** |
|  | Priority | Mean rank | 4.0 | 3.5 | | 4.3 | | 4.3 | *** |
| Access to healthcare providers | Satisfaction | Mean | 3.2 | 3.2 | | 3.3 | | 3.0 | NS |
|  | Priority | Mean rank | 4.2 | 4.1 | | 4.3 | | 4.3 | NS |
| Quality of infrastructure and services | Satisfaction | Mean | 3.2 | 3.3 | | 3.6 | | 2.7 | ** |
|  | Priority | Mean rank | 4.7 | 4.7 | | 4.5 | | 4.9 | NS |
| Reputation of healthcare providers | Satisfaction | Mean | 4.1 | 4.4 | | 4.1 | | 3.9 | NS |
|  | Priority | Mean rank | 4.7 | 4.8 | | 4.6 | | 4.8 | NS |
| Recommendation of the place of care by healthcare professionals | Satisfaction | Mean | 2.8 | 2.6 | | 3.4 | | 2.5 | * |
|  | Priority | Mean rank | 5.2 | 5.5 | | 5.1 | | 4.9 | * |
| Recommendation of the place of care by relatives or other patients | Satisfaction | Mean | 2.2 | 1.9 | | 2.7 | | 2.2 | * |
|  | Priority | Mean rank | 5.4 | 5.6 | | 5.4 | | 5.2 | ** |

Satisfaction is expressed in mean (range 0-5, 0 meaning not satisfied). Priority is expressed in mean rank (range 1-6, 1 meaning most important criterion). NS not significant; *p < 0.10; **p < 0.05; ***p < 0.01 by Kruskal-Wallis test

**Supplementary Table 2: Respondents’ satisfaction and priorities regarding information and services**

|  | | | **Global**  **(n = 201)** | | **Type 1 diabetes**  **(n = 67)** | | **Heart failure**  **(n = 67)** | **Obesity**  **(n = 67)** | **p-value** |
| --- | --- | --- | --- | --- | --- | --- | --- | --- | --- |
| Information and practical advice | Satisfaction | Mean | 3.2 | 3.5 | | 3.1 | | 2.9 | NS |
|  | Priority | Mean rank | 2.8 | 3.0 | | 2.6 | | 2.8 | NS |
| Scientific news | Satisfaction | Mean | 2.6 | 2.8 | | 2.4 | | 2.5 | NS |
|  | Priority | Mean rank | 4.0 | 3.7 | | 4.0 | | 4.2 | NS |
| Lifestyle and dietary measures | Satisfaction | Mean | 2.6 | 2.8 | | 2.4 | | 2.5 | NS |
|  | Priority | Mean rank | 3.7 | 4.3 | | 4.1 | | 2.7 | *** |
| Connected medical devices | Satisfaction | Mean | 2.6 | 3.6 | | 2.3 | | 1.8 | *** |
|  | Priority | Mean rank | 3.8 | 2.7 | | 4.0 | | 4.7 | *** |
| Psychological support | Satisfaction | Mean | 2.1 | 2.1 | | 2.1 | | 2.1 | NS |
|  | Priority | Mean rank | 4.0 | 4.4 | | 4.2 | | 3.5 | ** |
| Websites and mobile applications | Satisfaction | Mean | 2.7 | 3.0 | | 2.4 | | 2.5 | NS |
|  | Priority | Mean rank | 4.7 | 4.7 | | 4.5 | | 4.7 | NS |
| Telemedicine | Satisfaction | Mean | 1.7 | 2.1 | | 1.8 | | 1.1 | ** |
|  | Priority | Mean rank | 4.9 | 5.3 | | 4.3 | | 5.1 | *** |
| Connected devices | Satisfaction | Mean | 2.1 | 2.6 | | 2.0 | | 1.9 | NS |
|  | Priority | Mean rank | 5.2 | 5.0 | | 5.3 | | 5.2 | NS |

Satisfaction is expressed in mean (range 0-5, 0 meaning not satisfied). Priority is expressed in mean rank (range 1-6, 1 meaning most important criterion). NS not significant; *p < 0.10; **p < 0.05; ***p < 0.01 by Kruskal-Wallis test

**Supplementary Table 3: Respondents’ perception and priorities regarding the impact of the chronic condition on quality of life**

|  | | | **Global**  **(n = 201)** | | **Type 1 diabetes**  **(n = 67)** | | **Heart failure**  **(n = 67)** | **Obesity**  **(n = 67)** | **p-value** |
| --- | --- | --- | --- | --- | --- | --- | --- | --- | --- |
| Daily mood | Impact | Mean | 3.5 | 3.3 | | 3.3 | | 3.8 | * |
|  | Priority | Mean rank | 3.3 | 3.0 | | 3.8 | | 3.1 | *** |
| Social and family life | Impact | Mean | 3.4 | 3.3 | | 3.5 | | 3.4 | NS |
|  | Priority | Mean rank | 3.6 | 3.7 | | 3.7 | | 3.3 | NS |
| Ability to do physical activities | Impact | Mean | 3.3 | 3.1 | | 3.3 | | 3.6 | NS |
|  | Priority | Mean rank | 3.4 | 3.4 | | 3.3 | | 3.4 | NS |
| Autonomy | Impact | Mean | 2.2 | 1.7 | | 2.6 | | 2.5 | *** |
|  | Priority | Mean rank | 3.7 | 4.2 | | 3.1 | | 3.7 | *** |
| Food choices | Impact | Mean | 3.4 | 3.6 | | 2.9 | | 3.7 | *** |
|  | Priority | Mean rank | 4.4 | 4.2 | | 4.4 | | 4.5 | NS |
| Love/sex life | Impact | Mean | 3.0 | 2.7 | | 3.2 | | 3.2 | NS |
|  | Priority | Mean rank | 4.6 | 4.6 | | 4.4 | | 4.7 | NS |
| Friends and family | Impact | Mean | 2.8 | 2.3 | | 2.9 | | 3.1 | ** |
|  | Priority | Mean rank | 5.2 | 5.3 | | 5.1 | | 5.2 | NS |
| Professional life | Impact | Mean | 3.3 | 3.1 | | 3.6 | | 3.2 | NS |
|  | Priority | Mean rank | 4.9 | 4.6 | | 5.2 | | 5.0 | NS |

Impact is expressed in mean (range 0-5, 0 meaning not impacted). Priority is expressed in mean rank (range 1-6, 1 meaning most important aspect to preserve). NS not significant; *p < 0.10; **p < 0.05; ***p < 0.01 by Kruskal-Wallis test

**Supplementary Table 4: The study’s main results**

|  | **Global**  **(n = 201)** | **Type 1 diabetes**  **(n = 67)** | **Heart failure**  **(n = 67)** | **Obesity**  **(n = 67)** |
| --- | --- | --- | --- | --- |
| **3 most important and least satisfactory items regarding medical care** | 1- Access to coordinated and multidisciplinary care  2- Availability and active listening from healthcare providers  3- Taking the patient’s opinion into account for the choice of treatment | 1- Availability and active listening from healthcare providers  2- Access to healthcare providers  3- Access to coordinated and multidisciplinary care | 1- Availability and active listening from healthcare providers  2- Access to coordinated and multidisciplinary care  3- Taking the patient’s opinion into account for the choice of treatment | 1- Access to coordinated and multidisciplinary care  2- Access to innovative drugs and medical devices  3- Taking the patient’s opinion into account for the choice of treatment |
| **3 most important and least satisfactory items in terms of information and services** | 1- Connected medical devices  2- Lifestyle and dietary measures  3- Psychological support | 1- Psychological support  2- Scientific news  3- Lifestyle and dietary measures | 1- Connected medical devices  2- Scientific news  3- Psychological support | 1- Lifestyle and dietary measures  2- Psychological support  3- Scientific news |
| **3 most important aspects of quality of life that patients fear their chronic condition will impact the most** | 1- Daily mood  2- Ability to do physical activities  3- Social and family life | 1- Daily mood  2- Ability to do physical activities  3- Social and family life | 1- Ability to do physical activities  2- Social and family life  3- Daily mood | 1- Daily mood  2- Ability to do physical activities  3- Social and family life |
