# Supplementary material for: Chronic patients’ satisfaction and priorities regarding medical care, information and services and quality of life: a French online patient community survey
Source: BMC Health Serv Res. 2020 Jun 5;20:511. doi: 10.1186/s12913-020-05373-5 (PMC7275411; doi:10.1186/s12913-020-05373-5)
Supplement: Supplementary file 2 — Additional file 2. [file 12913_2020_5373_MOESM2_ESM.pdf]

A. RESPONDENT PROFILE

1. **You are:**
   (Only 1 answer)
   - ☐ A man
   - ☐ A woman

2. **Year of birth:**
   [Year] **[End of survey if age <18 years old]**

3. **ZIP code:**
   [5 numbers]

4. **What is the highest degree you have ever obtained?** *(Only 1 answer)*
   This question allows us to better understand the needs of each patient based on his or her experience.

   - ☐ Certificate of Primary Education (CEP)
   - ☐ BEPC, Elementary Certificate, College Certificate
   - ☐ CAP, BEP or equivalent diploma
   - ☐ Baccalaureate, professional or technician's certificate, BEA, BEC, BEI, BEH, higher certificate
   - ☐ Capacity in law, DAEU, ESEU
   - ☐ BTS, DUT, Deug, Deust, diploma of social professions, health diploma of level bac+2, other diploma of equivalent level
   - ☐ Bachelor's degree, pro degree, nursing diploma since 2012, master's degree, other diploma of bac+3 or bac+4 level
   - ☐ Master's, DEA, DESS, a diploma from a "Grande Ecole" with 5 years of higher education (engineering, business, etc.), or another diploma of equivalent level.
   - ☐ Doctorate in medicine, pharmacy, dentistry, other diploma of equivalent level
   - ☐ Research doctorate (outside the medical profession)
   - ☐ Other [Specify]
   - ☐ None

5. **You are:**
   (Only 1 answer)
   - ☐ A patient affected by obesity
   - ☐ A patient affected by type 1 diabetes
   - ☐ A patient affected by heart failure
   - ☐ Not affected by any of these diseases **[END OF SURVEY]**

6. **In what year was your disease diagnosed?**
   [Year]

Questions Q7 à Q11 are only for patients affected by obesity.

7. **How much do you weight (kg)?**
   Ex : 81 kg
   [Numeric field]

8. **How tall are you (cm)?**
   Ex : 174 cm
   [Numeric field]

END OF SURVEY if BMI <= 30

9. **What treatment(s) are you currently undergoing for your obesity?**
   (Several answers possible)
   - ☐ Hygieno-dietary rules (choosing low-energy foods, drinking water, controlling portion sizes, etc.). **>Q11**
   - ☐ Adapted physical activity **>Q11**
   - ☐ Psychotherapy **>Q11**
   - ☐ Drug treatments **>Q11**
   - ☐ Other *[Specify]* **>Q11**
   - ☐ None

10. **Why aren't you taking any medication?**
    [Open field]

11. **Have you had or will you have surgery for your obesity (gastrectomy, bypass, biliopancreatic bypass, etc.)?**
    (Only 1 answer)
    - ☐ Yes, I benefited from an operation
    - ☐ Yes, an intervention is planned in the near future **>Q19**
    - ☐ No **>Q19**

Questions 12 and 13 are only for patients who have had surgery in the past.

12. **When was the last time you had surgery for your disease?**
    (Only 1 answer)
    - ☐ Less than 2 years
    - ☐ Between 2 and 5 years
    - ☐ Between 5 and 10 years
    - ☐ More than 10 years

13. **Are you satisfied with your post-operative follow-up?**
    Mark from 0 to 5 (0= not at all satisfied, 5 = fully satisfied)
    (Only 1 answer per line)

	0<br>*= not at all satisfied*	1	2	3	4	5<br>*= Fully satisfied*
My post-op follow-up is:	o	o	o	o	o	o

Questions 14 and 15 are only for patients affected by type 1 diabetes.

14. What treatment(s) are you currently taking for your type 1 diabetes?
(Several answers possible)
- ☐ Hygieno-dietary rules (do not skip meals, choose high-fiber foods, etc.). **>Q19**
- ☐ Adapted physical activity **>Q19**
- ☐ Insulin pen **>Q19**
- ☐ Insulin pump **>Q19**
- ☐ Other *[Specify]* **>Q19**
- ☐ None

15. Why aren't you taking any medication?
[Open field]

Questions 16 and 17 are only for patients affected by heart failure.

16. What is the impact of your heart failure on your physical activity?
This question allows us to determine the stage of severity of your heart failure.
(Only 1 answer)
- ☐ No limitation of regular physical activity
- ☐ Modest limitation of physical activity (comfortable at rest, but ordinary activity leads to fatigue, palpitations, dyspnea)
- ☐ Marked limitation of physical activity (comfortable at rest, but less physical activity than usual causes symptoms and objective signs of cardiac dysfunction)
- ☐ Severe limitation: symptoms present even at rest

17. What treatment(s) are you currently taking for your heart failure?
(Several answers possible)
- ☐ Hygieno-dietary rules (limit salt-rich foods, prefer a Mediterranean diet, etc.). **>Q19**
- ☐ Adapted physical activity **>Q19**
- ☐ Drug treatment (conversion enzyme inhibitor, beta-blocker, aldosterone antagonist, anticoagulant, etc.). **>Q19**
- ☐ Implantable cardiac resynchronization devices (pacemaker, implantable automatic defibrillator, etc.) **>Q19**
- ☐ Surgical treatment (myocardial revascularization, valve surgery, heart transplant, etc.) **>Q19**
- ☐ Other *[Specify]* **>Q19**
- ☐ None

18. Why aren't you taking any medication?
[Open field]

This question applies to all survey participants.

19. In which place(s) of care is your illness covered?
(Several answers possible)

- ☐ City medical practice / private practice
- ☐ Care structure grouping together several health professionals: nursing home, health center, health pole, etc.
- ☐ Hospital or clinic
- ☐ Specialized establishment: specialized cure, specialized obesity center, diabetology center, follow-up care and rehabilitation center, rest center, etc.
- ☐ Other *[Specify]*

B. Define health value from the patient's perspective and determine whether the current health care offer meets their expectations.

20. To what extent do you agree with the following statements related to the medical management of your illness?

Mark from 0 to 5 (0= totally disagree, 5 = totally agree)

(Only 1 answer per line)

Please answer the question for your primary support

	0 = *Totally disagree*	1	2	3	4	5 = *Totally agree*	Do not know
My place of care and my health professionals have a good reputation.	o	o	o	o	o	o	o
My family and friends or other patients have recommended my place of care and my health care professionals.	o	o	o	o	o	o	o
A health professional recommended my place of care.	o	o	o	o	o	o	o
I have easy access to health professionals (satisfactory medical density) and to my place of care (location, public transport, parking, etc.).	o	o	o	o	o	o	o
I am satisfied with the infrastructures and services to which I have access (medical equipment, meals, accommodation, cleanliness of the premises, etc.).	o	o	o	o	o	o	o
I have access to innovative treatments and medical devices for the management of my disease.	o	o	o	o	o	o	o
My opinion is taken into account when I wish to choose innovative treatments and medical devices for the management of my disease.	o	o	o	o	o	o	o
I have access to coordinated care (different health professionals make medical decisions in a concerted manner) and multidisciplinary care (nutritionist, specialized sports educator, social worker, etc.).	o		o	o	o	o	
The health professionals who take care of me are attentive and available (ease of making appointments, availability by phone, email, etc.).	o		o	o	o	o	o

21. When assessing your medical management, what are the five criteria that are most important to you?

(1 = this criterion is the most important to me, 5 = this criterion is the least important to me)
Randomized Items

- ☐ Reputation of healthcare providers
- ☐ Recommendation of the place of care by relatives or other patients
- ☐ Recommendation of the place of care by healthcare professionals
- ☐ Access to healthcare providers
- ☐ Quality of infrastructure and services
- ☐ Access to innovative drugs and medical devices
- ☐ Taking the patient's opinion into account for the choice of treatment
- ☐ Access to coordinated and multidisciplinary care
- ☐ Availability and active listening from healthcare providers

22. Are you part of a Therapeutic Education Programme (TEP, pharmaceutical interview)?

Therapeutic Education Programme are disease- and treatment-specific and have the main objectives of:

- *Inform the patient about his or her illness and treatment*
- *Learn how to administer treatment/use a medical device*
- *Minimize the risks associated with taking the treatment*
- *Promote proper follow-up of treatment (as prescribed by the doctor)*

(Only 1 answer)

- ☐ Yes
- ☐ No **>Q24**

23. Are you satisfied with your Therapeutic Education Program (TEP, Pharmaceutical interview)?

Mark from 0 to 5 (0= not at all satisfied, 5 = fully satisfied)
(Only 1 answer per line)

	0<br>*= not at all satisfied*	1	2	3	4	5<br>*= fully satisfied*
My Therapeutic Education Program	o		o	o	o	o

24. To what extent do you agree with the following statements related to information and services dedicated to your disease?
Mark from 0 to 5 (0= totally disagree, 5 = totally agree)
(Only 1 answer per line)

	0 *= Totally disagree*	1	2	3	4	5 *= Totally agree*	I do not have access to it
I am satisfied with the information and practical advice I have access to about my disease and its treatment.	o	o	o	o	o	o	o
I am satisfied with the information I have access to on scientific news (ongoing clinical trials, innovative medical treatments/devices, etc.).	o	o	o	o	o	o	o
I am satisfied with the health and diet and/or adapted sport programs to which I have access.	o	o	o	o	o	o	o
I am satisfied with the psychological support I have access to.	o	o	o	o	o	o	o
I am satisfied with the websites, mobile applications, tracking software dedicated to my disease to which I have access.	o	o	o	o	o	o	o
I am satisfied with the connected objects (connected watch, connected scale, etc.) I have access to.	o	o	o	o	o	o	o
I am satisfied with the connected medical devices (connected blood glucose meters, connected pacemakers/ pacemakers, connected blood pressure monitors) to which I have access.	o	o	o	o	o	o	o
I am satisfied with the telemedicine solutions (telemonitoring, hotline, online platform for healthcare professionals - patients, etc.) that I have access to.	o		o	o	o	o	o

25. **Which of the following five elements do you think are the most important to improve your daily life with the disease?**
(1 = this element is the most important to me, 5 = this element is the least important to me)
Randomized items
- ☐ Information and practical advice on my pathology and its treatments
- ☐ Information on scientific news (ongoing clinical trials, innovative medical treatments/devices, etc.).
- ☐ Hygieno-dietary and/or adapted sport programs
- ☐ Psychological support
- ☐ Websites, mobile applications, software dedicated to my disease
- ☐ Connected objects (connected watch, connected scale, etc.)
- ☐ Connected medical devices (connected blood glucose meter, connected pacemaker/pacemaker, connected blood pressure monitors)
- ☐ Telemedicine solutions (telemonitoring, hotline, online platform for healthcare professionals - patients, etc.).

26. **Do the medical devices you use (insulin pump, blood glucose meter, pacemakers/pacemakers, etc.) improve your daily life?**
(Only 1 answer)
- ☐ Not at all
- ☐ Very few
- ☐ Medium
- ☐ A lot
- ☐ I do not use medical devices

27. **To what extent do you agree with the following statements regarding the impact of your disease on your quality of life?**
Mark from 0 to 5 (0= totally disagree, 5 = totally agree)
(Only 1 answer per line)

	0 *= Totally disagree*	1	2	3	4	5 = *Totally agree*	Don't know/ irrelevant
Consequences on my professional life (work stoppages, obstacles to promotion, inability to work, etc.).	o	o	o	o	o	o	o
Consequences on my social and family life (frequency of outings, activities with family, friends, etc.).	o	o	o	o	o	o	o
Consequences on the life of those around me (mockery, days of absence, fatigue etc.)	o	o	o	o	o	o	o
Consequences on my intimate life (sexuality, libido, etc.)	o	o	o	o	o	o	o
Consequences on my daily morale (self-esteem, self-confidence, depression, mood swings, etc.).	o	o	o	o	o	o	o
Consequences on my autonomy at home (washing, moving around the house, cooking, etc.) or outside (difficulties in travelling and/or	o	o	o	o	o	o	o

taking public transport, transportation of treatment, etc.).							
Consequences on my ability to engage in normal physical activity (risk of hypoglycemia, shortness of breath, pain, etc.).	O		O	O	O	O	O
Consequences on my food choices (salt-free, sugar-free, low-fat diet, etc.)	O		O	O	O	O	O

28. What do you think will be the aspects of your life most affected by your disease in the next three years? Please detail the aspects that are most important to you.
[Open field]

29. Rank the five areas of your life where you want the disease to have the least impact.
(1 = this aspect of my life is the most important to me, 5 = this aspect of my life is the least important to me)
Randomized Items

- ☐ Professional life (work stoppages, barriers to promotion, inability to work, etc.)
- ☐ Social and family life (frequency of outings, activities with family, friends, etc.)
- ☐ Life of those around me (mockery, days of absence, fatigue, etc.)
- ☐ Intimate life (sexuality, libido, etc.)
- ☐ Moral in daily life (self-esteem, self-confidence, depression, mood swings, etc.).
- ☐ Autonomy at home (washing, moving around the house, cooking, etc.) or outside (difficulties travelling and/or taking public transport, transportation for treatment, etc.).
- ☐ Ability to engage in normal physical activity (risk of hypoglycemia, shortness of breath, pain, etc.).
- ☐ Dietary choices (salt-free, sugar-free, low-fat diet, etc.)

30. In this question we would like to identify the items of expenditure that you would like to see better reimbursed.
(1 = this expense item is adequately reimbursed, 10 = this expense item should be much better reimbursed)
(Only 1 answer per line)

	1 *= this expense item is adequately reimbursed*	2	3	4	5	6	7	8	9	10 *= this expense item should be much better reimbursed*	Irrelevant
Hospitalization costs	o	o	o	o	o	o	o	o	o	o	o
Medical consultations and examinations	o	o	o	o	o	o	o	o	o	o	o
Care in specialized establishments: specialized cure, specialized obesity center, diabetology center, etc.	o	o	o	o	o	o	o	o	o	o	o
Treatments	o	o	o	o	o	o	o	o	o	o	o
Medical devices	o	o	o	o	o	o	o	o	o	o	o
Loss of earnings due to work stoppages	o	o	o	o	o	o	o	o	o	o	o
Supportive care: dietician, psychologist, specialised sports educator, social worker, etc.	o	o	o	o	o	o	o	o	o	o	o
Home help services (home care nurse, housekeeping help, etc.)	o	o	o	o	o	o	o	o	o	o	o
Home adaptations, specific food, specific clothing, etc.	o	o	o	o	o	o	o	o	o	o	o
Medical transport	o	o	o	o	o	o	o	o	o	o	o

31. Did you give up expenses related to your disease because of the expensive cost?
Ex : Psychotherapist, Dietician, special diets, home furnishings, etc…
(Only 1 answer)
- ☐ Yes
- ☐ No **>Q33**

32. What expenses did you give up?
[Open field]

It is considered that patients will be offered coordinated care pathways that include care by several health professionals with key steps to be followed.

33. To what extent would you be interested in integrating this type of care pathway if it allowed for:
Mark from 1 to 10 (1 not interested at all, 10 = very interested)
(Only 1 answer per line)

	1 = not interested at all	2	3	4	5	6	7	8	9	10 = very interested
To reduce out-of-pocket costs	o	o	o	o	o	o	o	o	o	o
To simplify the organization of medical care	o	o	o	o	o	o	o	o	o	o
To improve the outcomes of treatments	o	o	o	o	o	o	o	o	o	o

C. Involvement of patients in the assessment of healthcare services

34. In your opinion, how could the opinion of patients be better taken into account by health authorities (evaluation of treatments, stages of treatment, level of reimbursement etc.)?
(Several possible answers)

- ☐ By discussing with health professionals about my care so that this information can be passed on to the health authorities.
- ☐ During ad hoc consultations (survey, poll, etc.) with a majority of patients
- ☐ By joining a patient association and thus integrating working groups with health industrialists and health authorities (evaluation of treatments, stages of treatment, level of reimbursement etc.).
- ☐ By following training to become a patient expert/referent and thus join working groups with health industrialists and health authorities (evaluation of treatments, stages of treatment, level of reimbursement etc.).
- ☐ Other *[Specify]*
- ☐ I don't think that patients' opinions should be taken into account by health authorities.

35. Personally, would you agree to:
(Several possible answers)

- ☐ Share with the health authorities the information resulting from your discussions with health professionals about your care**> END OK**
- ☐ Participate in training to become an expert/referent patient and thus join working groups with health industrialists and health authorities (evaluation of treatments, stages of treatment, level of reimbursement etc.) **> END OK**
- ☐ Join a patient association and thus integrate working groups with health industrialists and health authorities (evaluation of treatments, stages of treatment, level of reimbursement etc.) **> END OK**
- ☐ Participate in ad hoc consultations (survey, poll, etc.) with most patients **>END OK**
- ☐ Other *[Specify]* **> END OK**
- ☐ I don't agree to get personally involved

36. Why didn't you agree to get personally involved?
[Open field]
