# Supplementary material for: Chronic patients’ satisfaction and priorities regarding medical care, information and services and quality of life: a French online patient community survey
Source: BMC Health Serv Res. 2020 Jun 5;20:511. doi: 10.1186/s12913-020-05373-5 (PMC7275411; doi:10.1186/s12913-020-05373-5)
Supplement: Supplementary file 3 — Additional file 3. [file 12913_2020_5373_MOESM3_ESM.pdf]

A. PROFIL DES REPONDANTS

1. **Vous êtes :**
   (1 seule réponse possible)
   - ☐ Un homme
   - ☐ Une femme

2. **Votre année de naissance :**
   [Champ année] **[FIN ENQUETE si âge <18 ans]**

3. **Votre code postal :**
   [5 chiffres]

4. **Quel est le diplôme le plus élevé que vous ayez obtenu ?**
   (1 seule réponse possible)
   Cette question nous permet de mieux comprendre les besoins de chaque patient en fonction de son vécu
   - ☐ Certificat d'études primaires (CEP)
   - ☐ BEPC, brevet élémentaire, brevet des collèges
   - ☐ CAP, BEP ou diplôme de niveau équivalent
   - ☐ Baccalauréat, brevet professionnel ou de technicien, BEA, BEC, BEI, BEH, brevet supérieur
   - ☐ Capacité en droit, DAEU, ESEU
   - ☐ BTS, DUT, Deug, Deust, diplôme des professions sociales, diplôme de santé de niveau bac+2, autre diplôme de niveau équivalent
   - ☐ Licence, licence pro, diplôme d'infirmière depuis 2012, maîtrise, autre diplôme de niveau bac+3 ou bac+4
   - ☐ Master, DEA, DESS, diplôme de grande école de niveau bac+5 (ingénieur, commerce, …), autre diplôme de niveau équivalent
   - ☐ Doctorat en médecine, pharmacie, odontologie, autre diplôme de niveau équivalent
   - ☐ Doctorat de recherche (hors profession médicale)
   - ☐ Autre *[Précisez]*
   - ☐ Aucun

5. **Vous êtes :**
   (1 seule réponse possible)
   - ☐ Patient atteint d'obésité
   - ☐ Patient diabétique de type 1
   - ☐ Patient insuffisant cardiaque
   - ☐ Non concerné par l'une de ces maladies **[FIN D'ENQUETE]**

6. **En quelle année votre maladie a-t-elle été diagnostiquée ?**
   [Champ année]

Les questions Q7 à Q11 concernent uniquement les patients atteints d'obésité.

7. **Quel est votre poids (en kg) ?**
   Ex : 81 kg
   [Champ numérique]

8. **Quel est votre taille (en cm) ?**
   Ex : 174 cm
   [Champ numérique]

FIN D'ENQUETE si IMC <= 30

9. **Quel(s) traitement(s) suivez-vous actuellement pour votre obésité ?**
   (Plusieurs réponses possibles)
   - ☐ Règles hygiéno-diététiques (choisir des aliments de faible valeur énergétique, boire de l'eau, contrôler la taille des portions, etc.) **>Q11**
   - ☐ Activité physique adaptée **>Q11**
   - ☐ Psychothérapie **>Q11**
   - ☐ Traitements médicamenteux **>Q11**
   - ☐ Autre *[Précisez]* **>Q11**
   - ☐ Aucun

10. **Pourquoi ne suivez-vous pas de traitement ?**
    [Champ libre]

11. **Avez-vous bénéficié ou allez-vous bénéficié d'une opération chirurgicale pour votre obésité (gastrectomie, bypass, dérivation biliopancréatique, etc.) ?**
    (1 seule réponse possible)
    - ☐ Oui, j'ai bénéficié d'une opération
    - ☐ Oui, une intervention est prévue dans peu de temps **>Q19**
    - ☐ Non **>Q19**

Les questions 12 et 13 concernent uniquement les patients ayant bénéficié d'une opération dans le passé.

12. **A quand remonte la dernière opération pour votre maladie ?**
    (1 seule réponse possible)
    - ☐ Moins de 2 ans
    - ☐ Entre 2 et 5 ans
    - ☐ Entre 5 et 10 ans
    - ☐ Plus de 10 ans

13. **Etes-vous satisfait de votre suivi post-opératoire ?**
    Noter de 0 à 5 (0 = pas du tout satisfait, 5 = tout à fait satisfait)
    (1 seule réponse par ligne)

	0 *= pas du tout satisfait*	1	2	3	4	5 *= tout à fait satisfait*
Mon suivi post-opératoire est :	o	o	o	o	o	o

La question 14 et 15 concerne uniquement les patients diabétiques de type 1.

14. Quel(s) traitement(s) suivez-vous actuellement pour votre diabète de type 1 ?
(Plusieurs réponses possibles)
- ☐ Règles hygiéno-diététiques (ne pas sauter de repas, choisir des aliments riche en fibre, etc.) **>Q19**
- ☐ Activité physique adaptée**>Q19**
- ☐ Stylo à insuline **>Q19**
- ☐ Pompe à insuline**>Q19**
- ☐ Autre *[Précisez]* **>Q19**
- ☐ Aucun

15. Pourquoi ne suivez-vous pas de traitement ?
[Champ libre]

Les questions 16 et 17 concernent uniquement les patients insuffisants cardiaques

16. Quel est l'impact de votre insuffisance cardiaque sur votre activité physique ?
Cette question nous permet de déterminer le stade de gravité de votre insuffisance cardiaque.
(1 seule réponse possible)
- ☐ Aucune limitation de l'activité physique ordinaire
- ☐ Limitation modeste de l'activité physique (à l'aise au repos, mais l'activité ordinaire entraîne une fatigue, des palpitations, une dyspnée)
- ☐ Limitation marquée de l'activité physique (à l'aise au repos, mais une activité physique moindre qu'à l'accoutumée provoque des symptômes et des signes objectifs de dysfonction cardiaque)
- ☐ Limitation sévère : symptômes présents même au repos

17. Quel(s) traitement(s) suivez-vous actuellement pour votre insuffisance cardiaque ?
(Plusieurs réponses possibles)
- ☐ Règles hygiéno-diététiques (limiter des aliments riche en sel, préférer un régime méditerranéen, etc.) **>Q19**
- ☐ Activité physique adaptée **>Q19**
- ☐ Traitement médicamenteux (inhibiteur de l'enzyme de conversion, béta-bloquant, antagoniste de l'aldostérone, anticoagulant, etc.) **>Q19**
- ☐ Dispositifs implantables de resynchronisation cardiaque (pacemaker, défibrillateur automatique implantable, etc.) **>Q19**
- ☐ Traitement chirurgical (revascularisation myocardique, chirurgie valvulaire, transplantation cardiaque, etc.) **>Q19**
- ☐ Autre *[Précisez]* **>Q19**
- ☐ Aucun

18. Pourquoi ne suivez-vous pas de traitement ?
[Champ libre]

Cette question s'applique à l'ensemble des participants de l'enquête

19. Dans quel(s) lieu(x) de soins votre maladie est-elle prise en charge ?
(Plusieurs réponses possibles)

- ☐ Cabinet médical de ville / libéral
- ☐ Structure de soins regroupant plusieurs professionnels de santé : maison de santé, centre de santé, pôle de santé, etc.
- ☐ Hôpital ou clinique
- ☐ Etablissement spécialisé : cure spécialisée, centre spécialisé de l'obésité, centre de diabétologie, centre de soins de suite et de réadaptation, centre de repos, etc.
- ☐ Autre *[Précisez]*

B. Définir la valeur en santé du point de vue du patient et déterminer si l'offre de soins actuelle répond à ses attentes

20. Dans quelle mesure êtes-vous d'accord avec les affirmations suivantes en lien avec la prise en charge médicale de votre maladie ?

Noter de 0 à 5 (0 = pas du tout d'accord 5 = tout à fait d'accord)

(1 seule réponse par ligne)

Merci de répondre à la question pour votre prise en charge principale

	0 *= pas du tout d'accord*	1	2	3	4	5 *= tout à fait d'accord*	Ne sait pas
Mon lieu de prise en charge et mes professionnels de santé ont une bonne réputation	o	o	o	o	o	o	o
Mon entourage ou d'autres patients m'ont recommandé mon lieu de prise en charge et mes professionnels de santé	o	o	o	o	o	o	o
Un professionnel de santé m'a recommandé mon lieu de prise en charge	o	o	o	o	o	o	o
J'ai facilement accès à des professionnels de santé (densité médicale satisfaisante) et à mon lieu de prise en charge (localisation, transports en commun, parking, etc.)	o	o	o	o	o	o	o
Je suis satisfait des infrastructures et des services auxquels j'ai accès (matériel médical, repas, hébergement, propreté des locaux, etc.)	o	o	o	o	o	o	o
J'ai accès à des traitements et des dispositifs médicaux innovants pour la prise en charge de ma maladie	o	o	o	o	o	o	o
Mon avis est pris en compte lorsque je souhaite choisir des traitements et des dispositifs médicaux innovants pour la prise en charge de ma maladie	o	o	o	o	o	o	o
J'ai accès à une prise en charge coordonnée (différents professionnels de santé prennent les décisions médicales de manière concertée) et multidisciplinaire (nutritionniste, éducateur sportif spécialisé, assistante sociale, etc.)	o		o	o	o	o	
Les professionnels de santé qui me prennent en charge sont à l'écoute et disponibles (facilité de prise de rendez-vous, disponibilité par téléphone, par mail, etc.)	o		o	o	o	o	o

21. **Lorsque vous évaluez votre prise en charge médicale, quels sont les cinq critères qui sont les plus importants pour vous ?**
(1 = ce critère est le plus important à mes yeux, 5 = ce critère est le moins important à mes yeux)
Items randomisés

- ☐ Réputation du lieu de prise en charge et de mes professionnels de santé
- ☐ Recommandation du lieu de prise en charge et de mes professionnels de santé par mon entourage ou d'autres patients
- ☐ Recommandation du lieu de prise en charge par un professionnel de santé
- ☐ Facilité d'accès à des professionnels de santé (densité médicale satisfaisante) et au lieu de prise en charge (localisation, transports en commun, parking, etc.)
- ☐ Qualité des infrastructures et des services (matériel médical de qualité, repas, hébergement, propreté des locaux, etc.)
- ☐ Accès à des traitements et à des dispositifs médicaux innovants pour la prise en charge de ma maladie
- ☐ Prise en compte de mes choix de traitements et de dispositifs médicaux innovants pour la prise en charge de ma maladie
- ☐ Accès à une prise en charge coordonnée (différents professionnels de santé prennent les décisions médicales de manière concertée) et multidisciplinaire (nutritionniste, éducateur sportif spécialisé, assistante sociale, etc.)
- ☐ Qualité de l'écoute et disponibilité des professionnels de santé (facilité de prise de rendez-vous, disponibilité par téléphone, par mail, etc.)

22. **Faites-vous parti d'un Programme d'Education Thérapeutique (ETP, entretien pharmaceutique) ?**
Les Programmes d'Education Thérapeutique sont spécifiques à une maladie et un traitement et ont pour principaux objectifs de :

- *Informer le patient sur sa maladie et son traitement*
- *Apprendre à administrer le traitement/utiliser un dispositif médical*
- *Minimiser les risques liés à la prise du traitement*
- *Favoriser le bon suivi du traitement (tel qu'il a été prescrit par le médecin)*

(1 seule réponse possible)

- ☐ Oui
- ☐ Non **>Q24**

23. Etes-vous satisfait de votre Programme d'Education Thérapeutique (ETP, entretien pharmaceutique) ?

Noter de 0 à 5 (0= pas du tout satisfait, 5 = tout à fait satisfait)

(1 seule réponse par ligne)

	0 *= pas du tout satisfait*	1	2	3	4	5 *= tout à fait satisfait*
Mon programme d'éducation thérapeutique	o		o	o	o	o

24. Dans quelle mesure êtes-vous d'accord avec les affirmations suivantes en lien avec l'information et les services dédiés à votre maladie ?

Noter de 0 à 5 (0 = pas du tout d'accord, 5 = tout à fait d'accord)

(1 seule réponse par ligne)

	0 *= pas du tout d'accord*	1	2	3	4	5 *= tout à fait d'accord*	Je n'y ai pas accès
Je suis satisfait de l'information et des conseils pratiques auxquels j'ai accès sur ma maladie et ses traitements	o	o	o	o	o	o	o
Je suis satisfait de l'information à laquelle j'ai accès sur l'actualité scientifique (essais cliniques en cours, traitements /dispositifs médicaux innovants, etc.)	o	o	o	o	o	o	o
Je suis satisfait des programmes hygiéno-diététiques et/ou de sport adapté auxquels j'ai accès	o	o	o	o	o	o	o
Je suis satisfait du soutien psychologique auquel j'ai accès	o	o	o	o	o	o	o
Je suis satisfait des sites Internet, des applications mobiles, logiciels de suivi dédiés à ma maladie auxquelles j'ai accès	o	o	o	o	o	o	o
Je suis satisfait des objets connectés (montre connectée, balance connectée, etc.) auxquels j'ai accès	o	o	o	o	o	o	o
Je suis satisfait des dispositifs médicaux connectés (lecteurs de glycémie connectés, pacemakers/ stimulateurs cardiaques connectés, tensiomètres connectés) auxquels j'ai accès	o	o	o	o	o	o	o
Je suis satisfait des solutions de télémédecine (télésurveillance, hotline, plateforme en ligne de professionnels de santé – patient, etc.) auxquelles j'ai accès	o		o	o	o	o	o

25. Parmi les éléments suivants, quels sont les cinq qui vous paraissent les plus importants pour améliorer votre quotidien avec la maladie ?

(1 = cet élément est le plus important à mes yeux, 5 = cet élément est le moins important à mes yeux)

Items randomisés

- ☐ Information et conseils pratique sur ma pathologie et ses traitements
- ☐ Information sur l'actualité scientifique (essais cliniques en cours, traitements/dispositifs médicaux innovants, etc.)
- ☐ Programmes hygiéno-diététiques et/ou de sport adapté
- ☐ Soutien psychologique
- ☐ Sites Internet, applications mobiles, logiciels dédiés à ma maladie
- ☐ Objets connectés (montre connectée, balance connectée, etc.)
- ☐ Dispositifs médicaux connectés (lecteur de glycémie connecté, pacemakers/stimulateurs cardiaques connectés, tensiomètres connectés)
- ☐ Solutions de télémédecine (télésurveillance, hotline, plateforme en ligne de professionnels de santé – patient, etc.)

26. Les dispositifs médicaux que vous utilisez (pompe à insuline, lecteur de glycémie, pacemakers/stimulateurs cardiaques, etc.) permettent-ils d'améliorer votre vie quotidienne ?

(1 seule réponse possible)

- ☐ Pas du tout
- ☐ Très peu
- ☐ Moyennement
- ☐ Beaucoup
- ☐ Je n'utilise pas de dispositifs médicaux

27. Dans quelle mesure êtes-vous d'accord avec les propositions suivantes en lien avec les conséquences de votre maladie sur votre qualité de vie :

Noter de 0 à 5 (0= pas du tout d'accord, 5 = tout à fait d'accord)

(1 seule réponse par ligne)

	0 *= pas du tout d'accord*	1	2	3	4	5 *= tout à fait d'accord*	Ne sait pas / non concerné
Conséquences sur ma vie professionnelle (arrêts de travail, freins à la promotion, impossibilité de travailler, etc.)	o	o	o	o	o	o	o
Conséquences sur ma vie sociale et familiale (fréquence des sorties, activités avec la famille, les amis, etc.)	o	o	o	o	o	o	o
Conséquences sur la vie mon entourage (moqueries, journées d'absences, fatigue etc.)	o	o	o	o	o	o	o
Conséquences sur ma vie intime (sexualité, libido, etc.)	o	o	o	o	o	o	o
Conséquences sur mon moral au quotidien (estime de soi, confiance en soi, dépression, sautes d'humeur, etc.)	o	o	o	o	o	o	o

Conséquences sur mon autonomie chez moi (se laver, se déplacer dans le domicile, faire à manger, etc.) ou à l'extérieur (difficultés à voyager et/ou à prendre les transports en commun, transport du traitement, etc.)	o	o	o	o	o	o	o
Conséquences sur ma capacité à pratiquer une activité physique normale (risque d'hypoglycémie, essoufflement, douleurs, etc.)	o		o	o	o	o	o
Conséquences sur mes choix alimentaires (régime sans sel, sans sucre, allégé en graisse, etc.)	o		o	o	o	o	o

28. D'après vous, quels seront les aspects de votre vie les plus impactés par votre maladie dans les trois prochaines années ? Merci de détailler les aspects qui ont le plus d'importance pour vous.
[Champ libre]

29. Classer les cinq aspects de votre vie pour lesquels vous souhaitez que la maladie ait le moins de conséquences.
(1 = cet aspect de ma vie est le plus important à mes yeux, 5 = cet aspect de ma vie est le moins important à mes yeux)
Items randomisés

- ☐ Vie professionnelle (arrêts de travail, freins à la promotion, impossibilité de travailler, etc.)
- ☐ Vie sociale et familiale (fréquence des sorties, activités avec la famille, les amis, etc.)
- ☐ Vie de mon entourage (moqueries, journées d'absences, fatigue etc.)
- ☐ Vie intime (sexualité, libido, etc.)
- ☐ Moral au quotidien (estime de soi, confiance en soi, dépression, sautes d'humeur, etc.)
- ☐ Autonomie chez soi (se laver, se déplacer dans le domicile, faire à manger, etc.) ou à l'extérieur (difficultés à voyager et/ou à prendre les transports en commun, transport du traitement, etc.)
- ☐ Capacité à pratiquer une activité physique normale (risque d'hypoglycémie, essoufflement, douleurs, etc.)
- ☐ Choix alimentaires (régime sans sel, sans sucre, allégé en graisse, etc.)

30. Dans cette question nous souhaitons identifier les postes de dépense que vous souhaiteriez voir mieux remboursés :

Noter de 1 à 10 (1 = ce poste de dépense est suffisamment remboursé, 10 = ce poste de dépense devrait être beaucoup mieux remboursé)

(1 seule réponse par ligne)

	1 *= ce poste de dépense est suffisamment remboursé*	2	3	4	5	6	7	8	9	10 *= ce poste de dépense devrait être beaucoup mieux remboursé*	Non concerné par ce poste de dépense
Les frais d'hospitalisation	o	o	o	o	o	o	o	o	o	o	o
Les consultations médicales et examens médicaux	o	o	o	o	o	o	o	o	o	o	o
La prise en charge dans les établissements spécialisés : cure spécialisée, centre spécialisé de l'obésité, centre de diabétologie, etc.	o	o	o	o	o	o	o	o	o	o	o
Les traitements	o	o	o	o	o	o	o	o	o	o	o
Les dispositifs médicaux	o	o	o	o	o	o	o	o	o	o	o
Manque à gagner liés aux arrêts de travail	o	o	o	o	o	o	o	o	o	o	o
Les soins de support : diététicien, psychologue, éducateur sportif spécialisé, assistante sociale, etc.	o	o	o	o	o	o	o	o	o	o	o
Les services d'aides à domicile (infirmier à domicile, aide pour le ménage, etc.)	o	o	o	o	o	o	o	o	o	o	o
Les aménagements du domicile, l'alimentation spécifique, les vêtements spécifiques	o	o	o	o	o	o	o	o	o	o	o
Les transports médicalisés	o	o	o	o	o	o	o	o	o	o	o

31. Avez-vous renoncé à des dépenses liées à votre maladie à cause des frais que cela engendraient pour vous ?

Ex : Psychothérapeute, Diététicien, alimentation spécifique, aménagement du domicile, etc…

(Une seule réponse possible)

- ☐ Oui
- ☐ Non **>Q33**

32. A quelles dépenses avez-vous renoncé ?

[Champ libre]

Intercalaire :
Il est envisagé de proposer aux patients des parcours de soins coordonnés qui comprennent une prise en charge par plusieurs professionnels de santé avec des étapes clés à respecter.

33. Dans quelle mesure seriez-vous intéressé pour intégrer ce type de parcours de soins s'il permettait :
Noter de 1 à 10 (1 = pas intéressé du tout, 10 = très intéressé)
(1 seule réponse par ligne)

	1 = pas intéressé du tout	2	3	4	5	6	7	8	9	10 = très intéressé
De diminuer les frais restant à votre charge	o	o	o	o	o	o	o	o	o	o
De simplifier l'organisation de votre prise en charge : prise des RDV, recherche d'un spécialiste, etc.	o	o	o	o	o	o	o	o	o	o
D'améliorer les résultats de votre traitement	o	o	o	o	o	o	o	o	o	o

C. Implication des patients dans l'évaluation de l'offre de soins

34. Selon vous, comment l'avis des patients pourrait être mieux pris en compte par les autorités de santé (évaluation des traitements, étapes de la prise en charge, niveau de remboursement etc.) ?

(Plusieurs réponses possibles)

- ☐ En échangeant avec les professionnels de santé au sujet de ma prise en charge pour que ces informations soit remontées aux autorités de santé
- ☐ Lors de consultations ponctuelles (sondage, enquête, etc.) auprès d'une majorité de patients
- ☐ En adhérant à une association de patients et ainsi intégrer des groupes de travail avec des industriels de la santé et les autorités de santé (évaluation des traitements, étapes de la prise en charge, niveau de remboursement etc.)
- ☐ En suivant une formation pour devenir patient expert/référent et ainsi intégrer des groupes de travail avec des industriels de la santé et les autorités de santé (évaluation des traitements, étapes de la prise en charge, niveau de remboursement etc.)
- ☐ Autre *[Précisez]*
- ☐ Je ne pense pas que l'avis des patients doit être pris en compte par les autorités de santé

35. Personnellement, seriez-vous d'accord pour :

(Plusieurs réponses possibles)

- ☐ Partager avec les autorités de santé les informations issues de vos échanges avec les professionnels de santé au sujet de votre prise en charge **>FIN OK**
- ☐ Participer à une formation pour devenir patient expert/référent et ainsi intégrer des groupes de travail avec des industriels de la santé et les autorités de santé (évaluation des traitements, étapes de la prise en charge, niveau de remboursement etc.) **>FIN OK**
- ☐ Adhérer à une association de patients et ainsi intégrer des groupes de travail avec des industriels de la santé et les autorités de santé (évaluation des traitements, étapes de la prise en charge, niveau de remboursement etc.) **>FIN OK**
- ☐ Participer à des consultations ponctuelles (sondage, enquête, etc.) auprès d'une majorité de patients **>FIN OK**
- ☐ Autre *[Précisez]* **>FIN OK**
- ☐ Je ne suis pas d'accord pour m'impliquer personnellement

36. Pourquoi n'êtes-vous pas d'accord pour vous impliquer personnellement ?

[Champ libre]

FIN ENQUETE
